# Risk perception and usage of non-occupational post-exposure prophylaxis among fisherfolk in Ggulwe parish on the shores of Lake Victoria in central Uganda

**DOI:** 10.3389/fpubh.2023.1116317

**Published:** 2023-11-08

**Authors:** Daraus Bahikire, Miisa Nanyingi, Christine Atuhairwe, Catherine Matama, Lesley Rose Ninsiima, Mudarshiru Bbuye

**Affiliations:** ^1^Faculty of Health Sciences, Uganda Martyrs University, Kampala, Uganda; ^2^School of Public Health, Makerere University College of Health Sciences, Kampala, Uganda; ^3^Ministry of Health, Kampala, Uganda

**Keywords:** post exposure prophylaxis, HIV, Uganda, quantitative data collection and analysis, qualitative data collection and analysis, fishing folks, perception, usage

## Abstract

**Background:**

The use of non-occupational post-exposure prophylaxis (nPEP) to prevent HIV acquisition among those exposed as an approach to HIV prevention has expanded in Uganda. Although there are increased efforts to avail nPEP services among most at-risk populations, the usage of nPEP medicines remains low. Therefore, this study examined the risk perception and usage of non-occupational post-exposure prophylaxis (nPEP) among fisherfolk in the Ggulwe fishing parish, Bussi sub-county, Wakiso district.

**Methods:**

A cross-sectional study among adults was carried out from October 2020 to January 2021 in Ggulwe parish, Bussi sub-county, Wakiso district, to examine the usage of nPEP and factors influencing the usage. Data were collected using semi-structured questionnaires, and key informants' interviews were conducted among healthcare providers and the local leadership. The quantitative data were summarized using bivariate and multivariate logistic regression, while the qualitative data were analyzed thematically to enrich the quantitative results.

**Results:**

Overall, 248 fisherfolk encountered an event that required the use of nPEP, and of these, 55/248 (22.2%) were able to use nPEP to prevent them from acquiring HIV. The usage of nPEP among adults in the Bussi sub-county, Wakiso district, was associated with not knowing that HIV can be prevented using nPEP medicines (AOR:0.1, 95% CI 0.03–0.36, *p* < 0.001), lack of knowledge of the existence of nPEP (AOR: 0.3, 95% CI 0.13–0.76, *p* = 0.01), the perception that nPEP can effectively prevent HIV infection after exposure (AOR 0.0586, 95% CI: 0.0177–0.1944, *p* < 0.001), and the community's opinion affecting the willingness to take nPEP (AOR 0.1924, 95% CI: 0.0380–0.9727, *p* = 0.0462).

**Conclusion:**

The usage of nPEP among fisherfolk was low (22.2%). The low usage of nPEP was associated with a lack of knowledge and awareness about nPEP. This effort to improve the usage of nPEP should include community sensitization and HIV infection prevention using nPEP to raise awareness about HIV infection exposures and the risk of HIV infection during non-occupational exposures.

## Introduction

Globally, 76 million people have acquired HIV, and 33 million people have died of HIV/AIDS since the beginning of the HIV epidemic. In addition, 38.0 million (31.6–44.5 million) people were living with HIV at the end of 2019. An estimated 0.7% (0.6–0.9%) of adults aged 15–49 years worldwide are living with HIV, although the burden of the epidemic continues to vary considerably between countries and regions (1). In the first years of the HIV epidemic, condom use was practically the only method available for preventing HIV transmission through sexual contact (2). In recent years, there has been considerable progress with alternative prevention methods, such as post-exposure prophylaxis (PEP) (1). HIV post-exposure prophylaxis (PEP), which is the use of antiretroviral medications for 28 days to prevent HIV acquisition after high-risk exposure, has long been available and is recommended by the WHO, especially among HIV high-risk populations (3).

HIV/AIDS is a leading cause of death in sub-Saharan Africa (SSA), accounting for 71% of the global burden of the infection (4). Different strategies for HIV prevention and control, including early diagnosis, the use of antiretroviral therapy (ART), and post-exposure prophylaxis [PEP], are of considerable interest (5). The Joint United Nations Programme on HIV and AIDS (UNAIDS) stated that HIV prevention must remain the cornerstone of the HIV response to achieve UNAIDS' Fast-Track Strategy to End AIDS by 2030 (6).

In the Uganda Population-based HIV Impact Assessment (2016–2017), the prevalence of HIV infection is lowest (0.2%) among the 15–19-year-old age group and highest (13.6%) among the 50–54-year-old age group (7). This indicates that although remarkable progress has been made in reducing the prevalence of HIV infection in Uganda, the rate of infection is still high. There is a relatively higher prevalence of HIV (10.8%) among the young people living in fishing communities of Lake Victoria (8) compared to the general population, where HIV prevalence among young people is 4.2% (7). The 2007 National Policy Guidelines on Post-Exposure Prophylaxis in Uganda for HIV, Hepatitis B, and Hepatitis C recommend a 28-day course within 36–72 h of exposure to HIV (9). As long as individuals continue to be exposed to HIV, there will be a role for PEP in the foreseeable future. Non-occupational PEP, the majority of which is for sexual exposure (PEPSE), has a significant role to play in HIV prevention efforts (10). Recently, there have been efforts to extend post-exposure prophylaxis among high HIV-risk groups such as female sex workers, fisherfolk communities, and men who have sex with men (MSM) to reduce the risk of HIV infection following occupational and non-occupational exposure (11). Research studies show a significant high demand for post-exposure prophylaxis following exposure to non-consensual sex, mainly among the high-risk population groups (11). In addition, a recent study done in rural Kenya and Uganda showed high retention and adherence to HIV post-exposure prophylaxis (PEP), contributing to the prevention of HIV (12). PEP has been included in the Uganda consolidated guidelines for the prevention and treatment of HIV and AIDS of 2020 (7).

The fisherfolk community is one of the high HIV-risk groups due to their frequent mobility, transactional and commercial sex, multiple sexual partners, high consumption of alcohol, poor health infrastructure, and limited access to health services (13–15). In addition to several interventions rolled out among the fisherfolk community to enhance HIV prevention and control, recent strategies have focused on extending non-occupational PEP in this community (16).

However, there is limited information on the factors associated with the usage of PEP in this community. Therefore, the study aimed to describe factors influencing the usage of non-occupational PEP in a fisherfolk community located on the shores of Lake Victoria in central Uganda.

## Materials and methods

### Study design and setting

**Figure 1:**
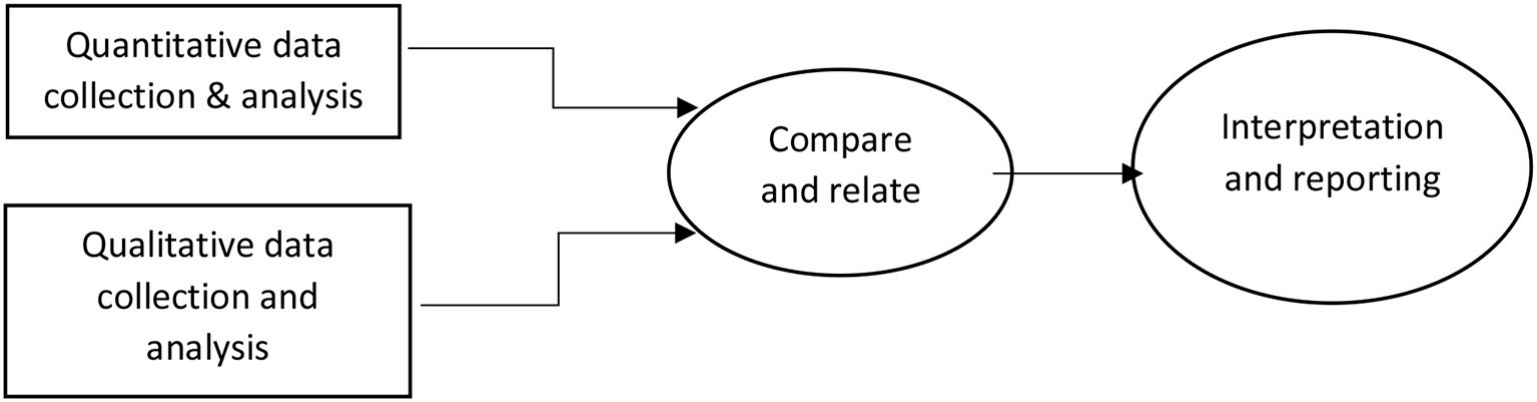
Diagrammatic representation of quantitative and qualitative approach.

This study used a cross-sectional study design employing both qualitative and quantitative methods of data collection (Figure 1). For the quantitative component, we used a structured questionnaire in which data were captured on exposure and usage of non-occupational PEP by persons who had been exposed to HIV. For the qualitative component, we held interviews with key informants and focus group discussions (FGDs) with the fisherfolk to enrich the quantitative results. Ggulwe fishing parish is a rural place, with a few trading centers found in the Bussi sub-county, Wakiso district, located in central Uganda. The population depends on fishing for a livelihood, with noticeable agriculture taking place. The area was selected for the study because the community is predominantly engaged in fishing and has a high (22%) HIV prevalence.

### Methodology implementation

#### Non-occupational prophylaxis usage in Uganda

In Uganda, HIV-PEP is provided to all eligible clients within 72 h of exposure. Non-occupational exposure is defined as non-occupational exposures including sexual assault (rape and defilement), road traffic accidents, unprotected sex with an HIV-infected person, and unprotected sex with a person of unknown HIV status (7, 17). The Ministry of Health (MOH) Uganda believes HIV infection can be aborted by inhibiting viral replication following an exposure of 48–72 h before the virus can be detected in the regional lymph nodes (10). Provision of nPEP in Uganda is provided for under the 2020 consolidated guidelines for the prevention and treatment of HIV/AIDS (10).

### Data source, study design, and population

A population of 5,011 spread across the five villages was used in the study based on the Uganda National Bureau of Statistics Population Census 2014. A cross-sectional mixed-method study was carried out among fisherfolk communities located along the shores of Lake Victoria in the Ggulwe parish, Wakiso district, from October 2020 to January 2021. To attain quantitative data, we included participants aged 15–49 years selected using purposive and simple random techniques, and for qualitative data, we used healthcare workers and local leaders due to their high level of knowledge on HIV prevention measures and interventions in the area. The sample size was determined using Krejcie and Morgan's (18) formula, and this gave us a sample size of 356 respondents.

### Measurements

Our outcome variable was the usage of non-occupational post-exposure prophylaxis, defined as receipt of the following: “Yes” for participants who had taken nPEP within 72 h of exposure and “No” for those who had not. The non-occupational exposure was measured by asking whether the participant had had sexual assault (rape and defilement), road traffic accidents, unprotected sex with an HIV-infected person, or unprotected sex with a person of unknown HIV status prior to the use of nPEP. The usage in this study was taken to be whether a participant utilized PEP obtained from either a government or private health facility after accidental or suspected exposure to HIV. The independent variables consisted of individual factors such as demographics, knowledge about PEP, risk perception, and knowledge of other HIV prevention measures; community-related factors including stigma, perceived effectiveness, and associated negative effects; and health facility-related factors consisting of attitude of health workers and availability of nPEP, and finally, nPEP knowledge among health workers and adherence to prescription guidelines.

### Data analysis

#### Quantitative data analysis

Data were analyzed using STATA version 14.0. Frequencies and their corresponding percentages were used for categorical variables, and mean and standard deviation were used for continuous variables that were normally distributed. The dependent variable was modeled as a binary outcome. Bivariate and multivariable analyses were performed using logistic regression with odds ratios as a measure of association. All variables with a *p* < 0.02 at bivariate analysis were included in the final multivariable model. Factors specified as important based on previous literature were included in the final model. A two-sided significance *p* < 0.05 and a 95% confidence interval were considered statistically significant for the analysis. Measures of association were reported as crude odds ratios at bivariate analysis and adjusted odds ratios at multivariable analysis.

#### Qualitative data analysis

To enrich and triangulate the quantitative results, we conducted qualitative interviews with men and women in Ggulwe fishing village, Bussi sub-county, Wakiso district. In particular, we held three FGDs, each consisting of six to eight people who were selected randomly from among those community members while maintaining COVID-19 SOPs. The group consisted of both men and women who had encountered or not encountered a situation that exposed them to HIV in the 12 months prior to the study. The FGDs were held within the village meeting rounds in the local language, “Luganda,” by two research assistants (DH and MN), both students trained in qualitative research methods. One research assistant (DH) moderated all the FGDs, while the other (MN) audio-recorded the responses and probed where necessary. Each FGD lasted for ~45 min on average. The moderator encouraged all the group members to ask questions and to provide comments as much as possible on HIV-PEP, knowledge, and usage. For key informant interviews (KIIs), four healthcare providers engaged in the provision of PEP services were purposefully selected and interviewed to elicit their expert opinions on the usage of PEP for HIV prevention. Both FGDs and KIIs were held until saturation was reached. Codes, subthemes, and themes were developed using NVivo V12 Pro, where the transcripts were uploaded and exported to Excel, which helped develop results.

#### Ethical considerations

The study obtained ethical approval from the research and ethics committee of Uganda Martyrs University. Administrative permission was sought from Ggulwe Parish. We obtained written informed consent from all the eligible participants who were above 18 years. Permission to include participants below the age of 18 was obtained from one or both of the parents/guardians depending on their accessibility. We then sought the child's assent to participate in the study. Those who declined to participate despite permission from their parents/guardians were not included in the study. The study obtained written informed consent from the adult participants. Participants under 18 years, who are considered emancipated minors, had to provide assent. Participation was voluntary and the study ensured maximum confidentiality considering the intricacy of the study topic. Furthermore, the team was mindful of the anticipated emotional discomfort from the survivors of rape and defilement. In such cases, psychosocial support in the form of confidential counseling and post-trauma support was offered by trained healthcare workers who were members of the study team, and further referrals where required were directed to the available community services.

## Results

### Sociodemographic characteristics

Table 1 shows that a total of 356 adults were selected and participated in the study. The majority of the participants [162 (45.5%)] interviewed were of the age group between 20 and 30 years, 148 (41.6%) were married or cohabiting, and more than half [185 (52.0%)] had attained a primary level of education. The majority were fishermen/mongers [134 (37.6%)], housewives [55 (15.4%)], peasants [14 (3.9%)], business persons [89 (25.0%)], casual laborers [32 (9.0%)], and others [32 (9.0%)] (Table 1). The results show that half of the participants were female [180 (50.6%)].

**Table 1:** Sociodemographic characteristics (*n* = 356).

**Category**	**All 356, n (%)**
**Sex**	
Male	176 (49.4)
Female	180 (50.6)
**Adult age group**	
15–20 years	34 (9.6)
20–30 years	162 (45.5)
30–40 years	116 (32.6)
>40 years	44 (12.4)
**Level of education**	
None	98 (27.5)
Primary	185 (52.0)
Secondary	60 (16.9)
Post-secondary	13 (3.7)
**Marital status**	
Single	144 (40.4)
Married	148 (41.6)
Separated	55 (15.4)
Widowed	9 (2.5)
**Occupation**	
Fisherman	134 (37.6)
Housewife	55 (15.4)
Peasant	14 (3.9)
Business person	89 (25.0)
Casual laborer	32 (9.0)
Other	32 (9.0)

### Usage of nPEP among fisherfolk in Ggulwe parish, Bussi sub-county, Wakiso district

Table 2 shows that overall, 248/356 (69.7%) adults encountered an event that required the use of nPEP, and of these, 55/248 (22.2%) were able to use nPEP to prevent them from acquiring HIV. The findings show that adults had experienced an event that required nPEP, and only 55 (22.2%) had utilized nPEP. Among the adults that encountered a situation that required nPEP, 17 (6.9%) had been raped, 196 (79.4%) had intercourse with partners whose HIV status was unknown, 17 (6.9%) had sexual intercourse with an HIV-infected person, and 17 (6.9%) had shared sharp objects with HIV-infected persons. Among the cases, the majority [146(59.1%)] reported that it had occurred once, 52 (21.1%) twice, 27 (10.9%) thrice, and 22(8.9%) said they were not sure. A relationship was found between the use of nPEP and being exposed once to HIV infection (OR 0.122, 95% CI 0.016–0.936, *p* = 0.044). Those with a single exposure are less likely to use PEP services. Being exposed multiple times to HIV infection was not statistically significant for nPEP usage.

**Table 2:** Situations associated with the usage of nPEP.

**Category**	**Usage of nPEP**		
	**No (%)**	**Yes (*n =* 55, %)**	**No (*n =* 193, %)**
**Were you in a situation that required the use of nPEP?**			
Yes	248	55 (22.2)	193 (77.8)
No	108 (30.3)		
**Type of situation**			
Was raped	17	15 (27.3)	2 (1.0)
Had sexual intercourse with a partner with unknown HIV status	197	36 (65.5)	160 (83.3)
Had sexual intercourse with an HIV-infected person	17	4 (7.3)	13 (6.8)
Shared sharp objects with infected persons	17	0 (0.0)	17 (6.9)
**The number of times this situation has occurred**			
Once	147	41 (74.5)	106 (54.9)
Twice	52	9 (16.4)	43 (22.4)
More than twice	27	4 (7.3)	23 (12.0)
Not certain	22	1 (1.8)	21 (10.9)

### Factors influencing uptake of non-occupational PEP

In the study, the assumption was that an individual who encountered a non-occupational event with an increased likelihood of HIV infection exposure used the non-occupational PEP. At multivariable analysis, the social demographic factors did not significantly influence the usage of non-occupational PEP. The study found that individuals who did not know that HIV infection could be prevented using nPEP were 90% less likely to use the nPEP services (AOR = 0.1, 95% CI = 0.03–0.36, *p* < 0.001). Similarly, individuals who did not know about non-occupational PEP were 70% less likely to use the services (AOR = 0.3, 95% CI = 0.13–0.76, *p* = 0.01). Furthermore, those individuals who did not know how non-occupational PEP was supposed to be used were less likely to use the services (AOR = 0.1, 95% CI = 0.03–0.19, *p* < 0.001). The results are summarized in Table 3. The results suggest that a lack of knowledge and awareness of non-occupational PEP is associated with a reduced likelihood of using the services.

**Table 3:** Factors influencing the uptake of non-occupational PEP.

**Category**	**Usage of nPEP**					
	**Yes (*n =* 55)**	**No (*n =* 193)**	**Crude odds ratio (95% CI)**	***p-*value**	**Adjusted Odds ratio (95% CI)**	***p-*value**
**Sex**						
Male	21 (38.2)	97 (50.3)	1.0			
Female	34 (61.8)	96 (49.7)	1.7 (0.91–3.08)	0.10	2.1 (0.56–7.78)	0.27
**Adult age group**						
15–20 years	6 (10.9)	17 (8.8)	1.0		1.0	
20–30 years	28 (50.9)	88 (45.6)	0.9 (0.32–2.50)	0.84	0.7 (0.15–2.91)	0.59
30–40 years	18 (32.7)	64 (33.2)	0.8 (0.27–2.31)	0.68	0.8 (0.17–4.07)	0.83
>40 years	3 (5.5)	24 (12.4)	0.4 (0.78–1.62)	0.18	0.3 (0.03–2.93)	030
**Level of education**						
None	12 (21.8)	52 (26.9)	1.0		1.0	
Primary	29 (52.7)	105 (54.4)	1.2 (0.57–2.55)	0.62	0.7 (0.24–2.18)	0.58
Secondary	9 (16.4)	33 (17.1)	1.2 (0.44–3.01)	0.78	0.7 (0.16–2.77)	0.58
Tertiary	5 (9.1)	3 (1.6)	7.2 (1.51–34.5)	**0.01**	1.3 (0.12–13.4)	0.83
**Marital status**						
Single	23 (41.8)	76 (39.4)	1.0			
Married	22 (40.0)	87 (45.0)	0.8 (0.43–1.62)	0.59	0.8 (0.29–2.01)	0.59
Separated	10 (18.2)	30 (15.5)	1.1 (0.47–2.59)	0.83	0.6 (0.16–2.12)	0.42
**Occupation**						
Business person	16 (29.1)	44 (22.8)	1.0		1.0	
Fisherman	15 (27.3)	75 (38.9)	0.6 (0.25–1.22)	0.14	1.1 (0.24–5.30)	0.87
Housewife	7 (12.7)	35 (18.1)	0.6 (0.20–1.48)	0.24	0.9 (1.99–3.76)	0.84
Peasant	10 (18.2)	20 (10.4)	1.4 (0.53–3.56)	0.51	1.4 (0.34–5.78)	0.64
Other	7 (12.7)	19 (9.8)	1.0 (0.36–2.86)	0.98	0.9 (0.18–4.98)	0.94
**Knowledge on nPEP**						
**Know that HIV can be prevented using nPEP**						
Yes	34 (61.8)	38 (19.7)	1.0		1.0	
No	7 (12.7)	87 (45.1)	0.1 (0.03–0.22)	**0.00**	0.1 (0.03–0.36)	**0.00**
Not sure	14 (25.5)	68 (35.2)	0.2 (0.11–0.49)	**0.00**	0.5 (0.18–1.43)	0.20
**Knew about nPEP**						
Yes	30 (54.5)	29 (15.0)	1.0		1.0	
No	25 (45.5)	164 (85.0)	0.2 (0.08–0.29)	**0.00**	0.3 (0.13–0.76)	0.01
**Knew how nPEP is taken**						
Yes	32 (58.2)	23 (11.9)	1.0		1.0	
No	16 (29.1)	164 (85.0)	0.1 (0.03–0.15)	**0.00**	0.1 (0.03–0.19)	**0.00**
Not sure	7 (12.7)	6 (3.1)	0.8 (0.25–2.83)	0.78	0.3 (0.05–1.23)	0.09
**Risk perception**						
**Can contract HIV, if extra care is not taken**						
Probably yes	18 (32.7)	44 (22.8)	1.0		1.0	
Most certainly	37 (67.3)	149 (77.2)	0.6 (0.32–1.17)	0.13	0.4 (0.17–1.05)	0.06
**Other Methods of HIV prevention**						
**Knew about HIV prevention methods**						
Yes	52 (94.5)	181 (93.8)	1.0		1.0	
No	3 (5.5)	12 (6.2)	0.9 (0.24–3.20)	0.83	0.8 (0.15–4.42)	0.80

95% confidence intervals for odds ratios (ORs) are in brackets. Bold indicates statistical significance.

### Results from the key informant interviews

The results from the key informant interviews show a similarity with the quantitative results, with the major themes influencing the uptake of non-occupational PEP.

It was observed that a lack of knowledge of the availability of non-occupational PEP may be hindering its usage among the Ggulwe fishing community in the Wakiso district. Regarding the knowledge of non-occupational PEP, the key informants reported that drugs do not reach the majority of people in the fishing community: one key informant explains in the quote below:

“*People are not aware of PEP. They are not aware of what can be done after a rape or after any other situation requiring PEP. Some immediately go to the LC leadership without knowing that the raped are supposed to be screened. Even one lady I sent to the health facility for PEP in August 2020 after the rape was not aware that she was supposed to receive PEP simply because she was not aware of it, yet the rapist was on ART. As per the interactions I have always heard, the community is not aware of PEP. I would say the community is at a 25% level of knowledge as regards PEP*” (Female, ART in-charge Bussi S/C).

Another participant cited that there were sensitization campaigns conducted by several organizations to educate communities about PEP.

“*UVRI did a lot of work to educate people but has never heard anyone say you can find that drug here or there. It is only UVRI that has done commendable work to educate the people about PEP in both Kava-enyanja and Kituufu sub islands but has since not heard of similar community awareness on the PEP. The awareness has not sufficiently sunk into the community. People don't know -how does it work; how does it help. There is a lot of awareness on HIV but it is lacking on PEP. People don't usually use it-I have never heard someone use it, yet as you see, this is an island with many prostitutes, so the awareness is lacking, it's not enough*” KI Councilor, Ggulwe parish.

### Knowledge of other HIV prevention measures

The various HIV/AIDS prevention methods cited were male circumcision, abstinence, the use of male condoms, avoiding multiple sexual partners, regular screening for HIV before having sex, being faithful to one sexual partner, and avoiding sharing sharp objects.

The study findings also revealed that the fishing community was given information and supplied with condoms. The health workers also encourage young people to abstain until marriage, although it proves fruitless. One of the key informants explained as follows:

“*The community knows other prevention but young girls are attracted by money to indulge in unprotected sex. We also give out free condoms but currently out of stock for 1 month. We can even spend 3 months without condoms, we tell clients to get condoms somewhere else or even from VHTs who usually have. On abstinence, people know – but it can't work here. Most of these people leave their families in Kampala and other areas, someone can't abstain from sex for a long period including the youths*,” KI enrolled nurse/M&E officer.

Following HIV preventive measures is futile in fishing communities since people get easy money and are driven to engage in risky sexual behavior.

“*Also, women in these communities are few and are shared among the available male. Again, there is a lot of alcoholism, a drunkard person can't easily have self-control on who to have or not to have sexual intercourse with, using barriers like condoms*,” KI Councilor, Ggulwe parish.

### Availability and attitude of healthcare providers

The following explanations were obtained. One key informant explained the following:

“*Even one lady I gave nPEP in August 2020 after the rape was not aware, she was supposed to receive PEP because she was not aware of it yet the rapist was on ART*,” KI, ART in-charge Bussi S/C.

From the arguments above, the indications are that little dissemination of WHO guidelines on administering PEP to clients exists. Most of the KIs reported that the community education programs of PEP are few, and they must educate clients on ART. One of the participants explained the following:

“*One may go to the health facility and find someone with no expertise in dealing with PEP, told to go back. The client may not go back for fear of being known by many people who may disclose one's status in public*,” KI, VHT coordinator, Bussi S/C.

The time spent while waiting to receive nPEP was found to be significant. The odds of an adult waiting <30 min to take nPEP were 0.05 times more likely than their counterparts that had not (OR 0.053, 95% CI. 0.004–0.696, *p* = 0.025). Other factors hindering nPEP service usage, such as fear of social stigma, distance to the health facility, refusal by a partner or spouse, and lack of knowledge about nPEP, were found to influence the usage of nPEP. Although 10/55(14.9%) of the clients reported that they had been denied nPEP at the health facilities in the Bussi sub-county, health workers denying clients nPEP because they may not qualify to take it were found to affect its use, and at times it was out of stock.

### Health facility-related factors

The key informant interviews revealed that the health workers can provide non-occupational PEP. However, there are hardly any health facilities where these services can be offered, especially to the rape victim. One of the participants explained the following:

“*Lack of access to the facility especially for kava-enyanja and Kituufu since there are no health centers in those areas, even no private facilities. There are only drug shops which I don't think offer PEP services*,” enrolled nurse.

Therefore, with the increase in health facilities, the health workers in the Ggulwe fishing community should be able to provide non-occupational PEP to their clients. In addition, regular health education on the uses of PEP during community outreach programs would not only increase knowledge but also raise awareness among rape victims to prevent them from contracting HIV infection.

### Myths and misconceptions about PEP

One key informant (HIV focal person of Bussi HC) said he gave it to someone who tested negative at the end of 3 months. However, one of the key informants explained the following:

“*Some say that once the health workers give you PEP medicine; you are already HIV positive. This is because it is HIV medicine. Differentiating between PEP and ARVs is a problem. This may be aggravated by disclosure to the partner about one's HIV status so that the negative partner can take PEP after sexual intercourse*,” KI enrolled nurse.

However, despite the community myths, most of the respondents did not have adequate knowledge of the use of non-occupational PEP in the prevention of HIV infection after exposure.

## Discussion

The study findings show that the level of knowledge on nPEP to prevent the acquisition of HIV was associated with the usage of nPEP. Conversely, while assessing the use of non-occupational PEP in MSM in the USA, Donnell et al. (19) found that 2,037 participants (47.5%) had heard of nPEP, with higher awareness reported at PEP sites (62%) relative to non-PEP sites (40%). nPEP sites had more recognition through advertising (23 vs. 8% at non-PEP sites) and newspapers (62 vs. 48%), whereas non-PEP sites had information from healthcare providers (18% at PEP sites vs. 29% at no-PEP sites) (19). In contrast, the knowledge of nPEP was low among the participants, given the widespread use of treatment for HIV/AIDS in Boston and San Francisco, USA with only 40% of sites without an active nPEP program. In addition, a certain study observed low knowledge of nPEP among young people in Nigeria (20). This implies that if a need arises for nPEP following a non-occupational exposure, many young people may not access services because they do not have the knowledge, thus potentially leading to new HIV infections that could have been averted. Despite the known and published benefits of PEP, this information is known to healthcare providers and policymakers but unavailable to the general public that is required to use this vital service (21).

The gap in knowledge about nPEP in the study area is not a special case as compared to the cited areas of Nigeria, Boston, and San Francisco. Similar approaches to enhance the uptake of this preventive therapy could be applied.

The perceived risk of acquiring HIV/AIDS was not significant in the non-occupational PEP. Similarly, in a study conducted in the USA among MSM, no association was found between the risk of HIV and nPEP use after exposure. Three seroconversions occurred at 384 visits (1.56 per 100 person-years) with nPEP use, compared to 210 seroconversions at 25,550 visits (1.64 per 100 person-years) with no nPEP use (hazard ratio: 0.91, 95% CI: 0.29, 2.86). The use of nPEP occurred more frequently in men with high-risk sexual behaviors. Those who had reported 10 or more partners had almost triple the adjusted odds of nPEP use (aOR 2.9, 95% CI 1.9, 4.4) relative to those who had reported zero to one (19). Significantly increased odds of nPEP use were seen in men with 2–5 and 6–9 partners. Previous studies in San Francisco showed that the odds of nPEP use increased with the HIV transmission of the reported risky sexual behaviors and low use of nPEP in the community setting (22), so the usage of nPEP is consistent with other cross-sectional studies in the USA, with only a small fraction of men reporting the use of nPEP after a high-risk exposure (23). The findings in other areas clearly agree with the study findings in Uganda. Despite the high risk of infection, communities remain reluctant to utilize PEP, and this clearly explains why HIV infections, notably in the fisherfolk communities, continue to soar.

Uganda is a culturally diverse country where culture and health behavior overlap, and it is important for researchers to assess how cultural beliefs and misconceptions might affect nPEP utilization across geographical regions of the country. The opinions of the community were found to influence the willingness of the community members to use nPEP. Many victims of rape would desire to use PEP but fear of being exposed (24). In contrast, a study done in South Africa among students revealed that they would take PEP if they thought they had been exposed to HIV infection, showing a positive attitude toward the use of nPEP (25). Similarly, Donnell et al. (19) established that individuals were willing to take nPEP if they perceived that they were at risk of acquiring HIV. Although there was no difference in willingness to use nPEP, at nPEP sites, 69% of MSM reported that they were very likely to use nPEP after a high-risk exposure, compared to 66% at non-nPEP sites (*p* = 0.06). A study on factors associated with the usage of nPEP among Thai men revealed that a higher proportion of them who intended to take nPEP answered “yes” when asked whether nPEP would reduce their concerns about becoming infected with HIV (86 vs. 65%, *p* < 0.001). There was no difference in the sense of stigmatization between those who did and those who did not intend to take nPEP (26).

This study revealed that participants would recommend the use of PEP to any potential client. PEP is one important means of controlling new infections, especially among people known to have been exposed (17), so making the population aware of the services is key for the prevention of new HIV infections. Conversely, during the qualitative interactions, most of the participants argued that the community should be educated on PEP.

The usage of non-occupational PEP can be increased through health education and promotion. A study by Kroon et al. (26) on the intent to use nPEP among Thai men revealed that participants who intended to take nPEP had more accurate knowledge about HIV transmission and prevention than those who did not. Approximately 90% of MSM in Thailand knew where to buy it and how soon after HIV exposure it should be taken. However, only 43% of those who recommended its use knew that it should be taken for 28 days (26).

## Study limitations

The study on risk perception and the usage of non-occupational post-exposure prophylaxis (PEP) among fisherfolk in Ggulwe parish on the shores of Lake Victoria in central Uganda had several limitations that need to be noted.

The LGBTQ concept was neither reported nor given the desired focus because there was no reference legal document with literature on the subject. This limited the extent to which this population was included and discussed in the study.

The sample size of the study was relatively small, which may have limited the generalizability of the findings to a broader population of fisherfolk in different regions. The limited scope of participants might have led to an underrepresentation of diverse perspectives and experiences within the fishing community.

The study heavily relied on self-reported data from the participants, introducing the potential for recall bias and social desirability bias. It is likely that participants provided responses that they believed were more socially acceptable, leading to an inaccurate representation of their actual risk perceptions and PEP usage.

Relatedly, due to resource constraints and logistical challenges, the study was conducted over a relatively short period of time. This timeframe might not have captured seasonal variations or long-term trends in risk perception and PEP usage among the fisherfolk. A more extended study period could have provided a more comprehensive understanding of this interesting area of study.

Furthermore, while efforts were made to ensure cultural sensitivity and local context adaptation, the study's design and data collection methods might not have fully captured the distinctions of the fisherfolk's beliefs, practices, and perceptions related to HIV prevention and PEP. Cultural factors that were not adequately accounted for could have influenced the results.

Finally, the researchers faced challenges in accessing some sub-island villages with limited health facilities, potentially leading to an underrepresentation of these areas in the study. This limitation might have affected the comprehensiveness of the findings and recommendations, particularly in terms of equitable PEP access.

## Conclusion

In conclusion, the 22% nPEP usage study sheds light on the critical need for comprehensive strategies to enhance the adoption and effective utilization of PEP within the fisherfolk communities of Ggulwe parish. The low usage was associated with limited awareness and knowledge about the non-occupational PEP.

There is a need to put together new strategies that publicize non-PEP in the general population, especially the sexually active age group.

There is a need to develop a collaborative approach that involves government bodies, beach management units, community-based organizations (CBOs), development partners, fisherfolk associations, local leaders, and village health teams (VHTs). This can be complemented by conducting health education outreach and employing peer educators from within the fisherfolk community.

Relatedly, the timely dissemination of consolidated guidelines for the prevention and treatment of HIV in Uganda is vital as a reliable resource for both healthcare providers and the fisherfolk. This information can immensely contribute to informed decision-making and reinforce the importance of PEP as an integral part of the overall strategy to combat HIV/AIDS.

Equally crucial is the need for equitable distribution of PEP within fisherfolk communities, especially in remote sub-island villages where health facilities are scarce. By extending PEP availability to the last mile, PEP will not only be accessible but will also be effectively utilized. This approach aligns with the overarching goal of leaving no one behind in the fight against HIV/AIDS.

Bottom-line, a community that understands the importance of PEP and its role in preventing HIV transmission is more likely to embrace the treatment and integrate it into their healthcare-seeking behaviors. By fostering a sense of ownership and empowerment within the fisherfolk community, the broader goal of eradicating AIDS becomes more achievable.

## Data availability statement

The datasets presented in this article are not readily available because date sets are restricted to the authors. Requests to access the datasets should be directed to dbahikire1990@gmail.com.

## Author contributions

DB conceived and designed the research study, collected data, wrote the initial draft of the manuscript, and responded to reviewers' comments. MN validated the data collection tools, supervised the study, provided guidance and general oversight during the entire course of the study, and also provided input in the review process. CA supported report compilation and performed statistical analyses. CM and LN contributed to the review of the manuscript. MB provided guidance and critically reviewed and revised the manuscript for important intellectual content. All authors contributed to the article and approved the submitted version.
