# Supplementary material for: Risk perception and usage of non-occupational post-exposure prophylaxis among fisherfolk in Ggulwe parish on the shores of Lake Victoria in central Uganda
Source: Front Public Health. 2023 Nov 8;11:1116317. doi: 10.3389/fpubh.2023.1116317 (PMC10663348; doi:10.3389/fpubh.2023.1116317)
Supplement: Supplementary file 1 [file Data_Sheet_1.pdf]

APPENDICES

Appendix i**: Questionnaire**

A. (i) INDIVIDUAL CHARACTERISTICS

1. Gender: 1-Male 2 -Female
2. Age: (years)----------------------------
   a) 15 – 20 years b) 20 –30 years c)30-40years d) 40 years & above

3. Level of education
   a) No formal education b) Primary c) Secondary d) Tertiary
4. Marital status
   a)-Single b) Married/Cohabiting c) Divorced/separated d) Widowed
5. Religion of the respondent
   a) Protestant b) Catholic c) Islam d) Other
6. Occupation
   a) Formal employment (gov't) b) Informal employment (private c)Unemployed

.

(ii) KNOWLEDGE ABOUT nPEP

7. Do you know that HIV infection can be prevented with medication?
   a) Yes b) No c) Unsure

8. Do you know nPEP?
   a) Yes b) No

9. Do you know how nPEP should be taken? a) Yes b) No c) Unsure

(iii) RISK PERCEPTION

10. Do you think you can contract HIV if you don't take preventive measures
    a) Certainly not b) Probably not c) Probably yes d) Most certainly
11. How serious/severe do you think is HIV/Aids?
    a) not at all serious b) not serious c) slightly serious d) very serious

(iv) KNOWLEDGE ON OTHER HIV PREVENTION MEASURES

12. Do you know other HIV prevention methods? a) Yes b) No
13. State the various HIV/Aids prevention methods

…………………………………………………………………………………………………
…………………………………………………………………………………………………
…………………………………………………………………………………………………

B. HEALTH FACILITY FACTORS

(i) ATTITUDE OF HEALTH WORKERS

14. Which situation in the last 6 months required you to take PEP
    a) Was raped
    b) Had sexual intercourse with a person I suspected to be infected
    c) Shared with a sharp object with a person I suspected was infected

15. How many times has this situation happened?
    a) Once
    b) Twice
    c) More than 2 times
16. Did you go for PEP
    a) Yes b) No

17. If Yes, where did you go for nPEP?
    a) Govt facility b) Private clinic/Drug store

(ii) AVAILABILITY OF PEP

18. Was PEP available
    a) Yes b) No

19. If yes, how did you access nPEP?
    a) For free b) Using money c) Unsure

(iii) KNOWLEDGE OF PEP AMONG HEALTH WORKERS

20. If you were able to get PEP, how long were you required to take the medication?
    a) One month
    b) Other specify…………………………………………………………....
21. Did you complete the course of treatment?
    a) yes
    b) No

(iv) ATTITUDE OF HEALTH WOKERS TOWARDS PEP CLIENTS
22. Were the health workers available when you visited a health facility?
    a) Yes b) No
23. Was the attitude of the health workers good? a) Yes b) No c) Unsure

24. Complete the table below

	Yes	No
a. Did the health workers provide information about who is mostly at risk?		
b. Did the health workers explain the legibility?		
c. Did the health workers explain when the treatment is most effective?		
d. Did the health workers explain the duration of treatment?		
e. Did the health workers explain when the HIV test can be taken after treatment?		

25. How long did you wait before receiving nPEP?

26. If you didn’t go for nPEP, what could be the reason?

a. Long waiting time to receive nPEP
b. Health facilities are few and far away
c. In adequate supply of nPEP
d. Poor response from the health workers
e. Refusal by partner(spouse)
f. Fear of social stigma
g. Poor quality of health service
h. Did not know that nPEP was accessible/available

27.Haye you ever been denied nPEP

a) Yes b) NO

28.What was the reason it was denied

a) Told I wasn’t eligible
b) Was out of stock
c) Others, specify

21. In your opinion, what do you think are the reasons why people don’t take nPEP health services
(**Multiple response**)

a) Long waiting time to receive nPEP
b) Health facilities are few and far away
c) In adequate supply of nPEP
d) Poor response from the health workers
e) Refusal by partner(spouse)
f) Fear of social stigma
g) Poor quality of health service
h) Did not know that nPEP was accessible/available /don’t know that PEP as medicine exists

SECTION C: COMMUNITY RELATED FACTORS

22. Have you ever heard people talking about PEP having side effects?
1-Yes 2-No 3-Unsure
23. If yes, could those side effects stop you from using PEP?
1-Yes 2-No 3-Unsure
24. Do You think PEP effectively prevents you from HIV infection after exposure?
1-Yes 2-No 3-Unsure
25. If No, State reasons……………………………….

26. What do people in the community say about PEP?

……………………………………………………………………………………………
……………………………………………………………………………………………

27. Do the community thoughts /opinions affect your willingness to take PEP?

1-Yes 2-No 3-Unsure

28.Would you recommend any member from the community to use PEP?

1-Yes 2-No 3-Unsure

29. If NO, why………………………………………………………………

Thank you for participating in this study

Appendix ii: **Key Informant Interview guide**

My name is **Bahikire Daraus**, a student of Master of Public Health-Health Promotion of Uganda Martyrs University. I am conducting a research on **"*Factors Influencing the Uptake of Post-Exposure Prophylaxis (PEP) Among Males and females 15-49 Years of Age in The Fishing Community of Ggulwe Parish, Bussi Sub county, Wakiso District, Central Uganda".*** I am conducting this research in partial fulfillment of the requirements for the award of Master of Public Health. Any information collected from this study will be kept confidential and will strictly be used for academic purposes. You have a right to choose to participate or not to participate in this study.

1. What is your opinion on nPEP as a preventive approach for HIV/AIDS?
2. What are the factors associated with the uptake of nPEP in the fishing community of Ggulwe Parish?
3. Are you aware of the guidelines regarding the use of nPEP? If any, please share
4. What is your opinion on nPEP availability in facilities?
5. In your opinion, what are the levels of community knowledge regarding nPEP?
6. How is the demand for nPEP in the fishing community of Ggulwe Parish?
7. What's your take on the health worker's attitude towards nPEP clients?
8. What are the community thoughts about nPEP?
9. What could be done to improve nPEP uptake in Ggulwe parish, Bussi county, Wakiso District?

Appendix iii **Focused group discussion guide**

My name is **Bahikire Daraus**, a student of Master of Public Health-Health Promotion of Uganda Martyrs University. I am conducting a research on **"*Factors Influencing the Uptake of Post-Exposure Prophylaxis (PEP) Among Males and females 15-49 Years of Age in The Fishing Community of Ggulwe Parish, Bussi Sub county, Wakiso District, Central Uganda".*** I am conducting this research in partial fulfillment of the requirements for the award of Master of Public Health. Any information collected from this study will be kept confidential and will strictly be used for academic purposes. You have a right to choose to participate or not to participate in this study.

1. What is your opinion on nPEP as a preventive approach for HIV/AIDS?

2. What are the factors affecting the uptake of nPEP in the fishing community of Ggulwe Parish?
3. Are you aware of the guidelines regarding the use of nPEP? If any, please share
4. What is your opinion on nPEP availability in facilities?
5. In your opinion, what are the levels of community knowledge regarding nPEP?
6. How is the demand for nPEP in the fishing community of Ggulwe Parish?
7. What's your take on the health worker's attitude towards nPEP clients?
8. What are the community thoughts about nPEP?
9. What could be done to improve nPEP uptake in Ggulwe parish, Bussi county, Wakiso District?

Thank you for participating in this study

Appendix iii: **Sampling guide**

TABLE FOR DETERMINING SAMPLE SIZE FROM A GIVEN POPULATION

N	S	N	S	N	S	N	S	N	S
10	10	100	80	280	162	800	260	2800	338
15	14	110	86	290	165	850	265	3000	341
20	19	120	92	300	169	900	269	3500	246
25	24	130	97	320	175	950	274	4000	351
30	28	140	103	340	181	1000	278	4500	351
35	32	150	108	360	186	1100	285	5000	357
40	36	160	113	380	181	1200	291	6000	361
45	40	180	118	400	196	1300	297	7000	364
50	44	190	123	420	201	1400	302	8000	367
55	48	200	127	440	205	1500	306	9000	368
60	52	210	132	460	210	1600	310	10000	373
65	56	220	136	480	214	1700	313	15000	375
70	59	230	140	500	217	1800	317	20000	377
75	63	240	144	550	225	1900	320	30000	379
80	66	250	148	600	234	2000	322	40000	380
85	70	260	152	650	242	2200	327	50000	381
90	73	270	155	700	248	2400	331	75000	382
95	76	270	159	750	256	2600	335	100000	384

Note: "N" is population size
"S" is sample size.

Krejcie, Robert V., Morgan, Daryle W., "Determining Sample Size for Research Activities", Educational and Psychological Measurement, 1970.
